# Drug Delivery Systems Obtained from Silica Based Organic-Inorganic Hybrids

**DOI:** 10.3390/polym8040091

**Published:** 2016-03-24

**Authors:** João Augusto Oshiro Junior, Marina Paiva Abuçafy, Eloísa Berbel Manaia, Bruna Lallo da Silva, Bruna Galdorfini Chiari-Andréo, Leila Aparecida Chiavacci

**Affiliations:** 1 Faculdade de Ciências Farmacêuticas, UNESP—Univ Estadual Paulista, Araraquara-Jaú, Km 1, Araraquara 14800-903, Brazil; joaooshiro@yahoo.com.br (J.A.O.J.); marina.abucafy@gmail.com (M.P.A.); elobm_elo@yahoo.com.br (E.B.M.); brunalallo@hotmail.com (B.L.S.); brunagchiari@yahoo.com.br (B.G.C.-A.); 2 Departamento de Ciências Biológicas e da Saúde, Centro Universitário de Araraquara—UNIARA, Araraquara 14800-903, Brazil

**Keywords:** organic-inorganic materials, drug delivery systems, sol-gel process

## Abstract

This is a review of hybrid materials based on silica as an inorganic phase used as drug delivery systems (DDS). Silica based DDS have shown effectivity when compared with traditional delivery systems. They present advantages such as: (a) ability to maintain the therapeutic range with minor variations; (b) prevention of local and systemic toxic effects; (c) plasma concentrations increase of substances with a short half-life; and (d) reduction of the number of daily doses, which may increase patient adherence to the treatment. These advantages occur due to the physical, chemical and optical properties of these materials. Therefore, we discuss the properties and characteristics of them and we present some applications, using different approaches of DDS to ensure therapeutic effectiveness and side effects reduction such as implantable biomaterial, film-forming materials, stimuli-responsive systems and others.

## 1. Introduction: Hybrid Materials in Drug Delivery

A drug administration is considered successful when it ensures therapeutic effectiveness, minimizes the occurrence of side effects and does not introduce unacceptable levels of toxicity [1,2]. This can be achieved by discovering new drugs as well as by administrating existent drugs through new delivery devices, as controlled release systems. These systems stand out because they have several advantages over conventional methods of drug administration such as: the ability to incorporate lipophilic and hydrophilic substances, lower toxicity, prolonged permanence in the bloodstream, gradual and controlled drug release and safe administration (do not offer local inflammatory response). They also allow the use of suitable dosage and targeting the drug to specific sites [3,4,5,6].

The use of polymeric materials has attracted attention in the development of such drug controlled release devices due to their high capacity of processing and physical-chemical properties adapted through synthesis [7,8]. Thus, a wide variety of polymers have been tested in drug delivery devices, being cellulose derivatives and the polymeric matrices based on poly(ethylene oxide) (PEO) are the most used [9]. However, PEO is widely used in the pharmaceutical industry, due to low toxicity, high capacity for swelling (hydrophilic character) and stability in the biological environment pH [10].

In contrast, poly(propylene oxide) (PPO) has an additional methyl group giving to this polyether a more hydrophobic character than PEO. The combination of hydrophilic-hydrophobic character of these macromolecules is quite exploited for drug delivery systems [11,12,13]. An example is the block copolymers, commercially known as Pluronic^®^, which are formed by blocks of PEO and PPO arranged on the basic structures OEx-OPy-OEx (usually abbreviated as PEO-PPO-PEO) [10,14,15]. In these polymers, the control of drug release depends on the solubility, drug particle size and the polymer viscosity. When the release medium (e.g., water) is thermodynamically compatible with the polymer, it can occur a relaxation process of the polymer chain, which becomes more flexible, causing swelling and facilitating the diffusion of the drug out of the matrix. Besides, the water-soluble polymer (PEO) allows the erosion of matrix, which is another important factor in controlling the release rate. Thus, the release mechanisms from hydrophilic matrices can be explained by the complex relationship between the swelling, diffusion and erosion of matrix [16].

Numerous drug delivery systems (DDS) have been developed with biologically compatible polymers [17,18,19,20] but not always their properties (mechanical, physical, chemical and optical) allow the development of multifunctional devices (subcutaneous implants, occlusive bandages and films) suitable for both controlled target and drug release. Therefore, the development of nanotechnology has provided the achievement of nanostructured polymers, which are an alternative to conventional polymeric systems. The nanometric modification of the structure of these materials offers optimized structural, optical and mechanical characteristics, besides others suitable characteristics for application in the controlled drug delivery. Among this new class of nanostructured polymers, the organic-inorganic hybrid materials, also known as nanocomposites, are highlighted [21].

The use of the term “organic-inorganic hybrid materials” began in the last 35 years. The development of this area has been accelerated since the 80s, especially for the preparation of inorganic gels, impregnated by organic polymers [22]. These organic-inorganic hybrid materials combine synergistically the physicochemical characteristics of their constituents, allowing the achievement of unique properties, making this class of materials promising candidates for the development of new multifunctional systems with wide applications [23,24,25,26]. The organic phase provides specific physical and/or chemical properties (optical, electrical, reactivity), while the inorganic phase increases the mechanical strength, thermal stability, allows to modulate the refractive index, and favors the rheological properties of the final material, depending on its shapes and sizes [27,28,29].

Organic-inorganic hybrid materials are nanocomposites in which occurs the interpenetration of the two phases in a nanometer scale [30]. When the phases have nanometric dimensions (1–500 nm) they exhibit a high surface area, promoting better dispersion in the polymer matrix. This phenomenon improves the physical characteristics of the composite that depend on the homogeneity of the material [31,32]. The nature of the organic-inorganic interface is used to define three classes of hybrid materials [33]: class I is that which exhibit weak bonds between the two phases (van der Waals bonds, hydrogen bonds or electrostatic bonds); Class II presents strong bonds between the two phases (covalent or ionic bonds); Class III presents a combination of interactions that occurring in both class I and II.

In the class I hybrid materials, the process of preparation involves the addition of non-polymerizable organic molecular precursors, which are soluble in the medium that is obtained in pure silica; however, it does not participate directly in the reaction of gelation [33]. In the Class II hybrids, polymerizable organosilanes are used as precursors of organic component that have organic group bonded directly to silicon (Si–C non-hydrolyzing). Hybrids of this class exhibit greater thermal stability when compared to the organic component of class I [34,35,36].

**Figure 1:**
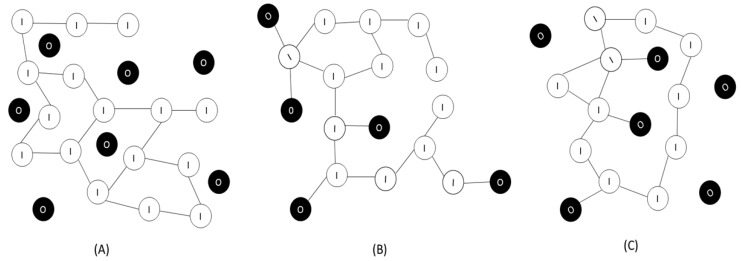
Representation of (**A**) Class I; (**B**) Class II; and (**C**) Class III of organic-inorganic hybrid materials. The white symbols represent the inorganic phase and the black ones represent the organic phase (adapted from the study by Benvenutti *et al.*, 2009 [33]).

The Figure 1 shows a representative scheme of different classes of organic-inorganic hybrid materials.

Recent reviews provide more focused overviews about the role of nanostructure of different materials in biological applications, thereby we will not deepen this discussion (for reviews, see [37,38]).

Several hybrid materials are synthesized and processed from of a chemical route called sol-gel. In this route, an organosilane precursor is polymerized in a matrix of organic macromolecules. The sol term is used to define a dispersion of colloidal particles (size between 1 to 999 nm) stabilized in a fluid, while the gel term is used to refer to a system formed by the rigid structure of colloidal particles (colloidal gel) or polymer chains (polymer gel) that immobilizes the liquid phase in the interstices [39]. This process is based on: (a) copolymerization of functional organosilanes, metal alkoxides and macromonomers; (b) encapsulation of organic compounds based in silica or metal alkoxides; (c) functionalization of nanoparticles, nanoclays or other compounds with lamellar structures, *etc.* The use of sol-gel process in the preparation of these new materials allows the production of gels (which can be used in masks or occlusive dressings), thin films, powders and microspheres at room temperature. The hybrid materials composed by the silica as inorganic component are attractive for technological applications, mainly due to the high stability of the Si–C bond. Moreover, Si–O–Si networks are important in terms of transparency, thermal stability, and mechanical strength [40,41]. In addition, these silica-based hybrid materials exhibit biocompatibility [42].

The chemical reactions involved in a conventional sol-gel process, based on alkoxides derivatives are: hydrolysis (step 1), where the OR groups are replaced by silanol groups (Si–OH); subsequently, these silanol groups can react with each other (step 2), or with other groups OR (step 3) via condensation reactions by forming siloxane bonds, giving a three-dimensional network of silica [33,39].

SiOR + H_2_O → SiOH + ROH (step 1)

SiOH + SiOH → SiOSi + H_2_O (step 2)

SiOH + SiOR → SiOSi + ROH(step 3)

A great control of this gelling process could be reached with these hybrids. This is due to the silicon precursor, which decreases the rate of gelling reaction [39]. Thus, it is possible to modulate the final properties of materials such as size and shape of particles, volume and pore size distribution [33]. Due to these unique characteristics, the silica-based materials have applications in several areas such as adsorbent for dye, electronics, optics, mechanics, energy, environment, biology and medicine. In these fields, silica-based materials have been used as membranes and separation devices, solar cells, catalysts and sensors, drug carriers, among others [43,44,45,46,47,48,49].

**Figure 2:**
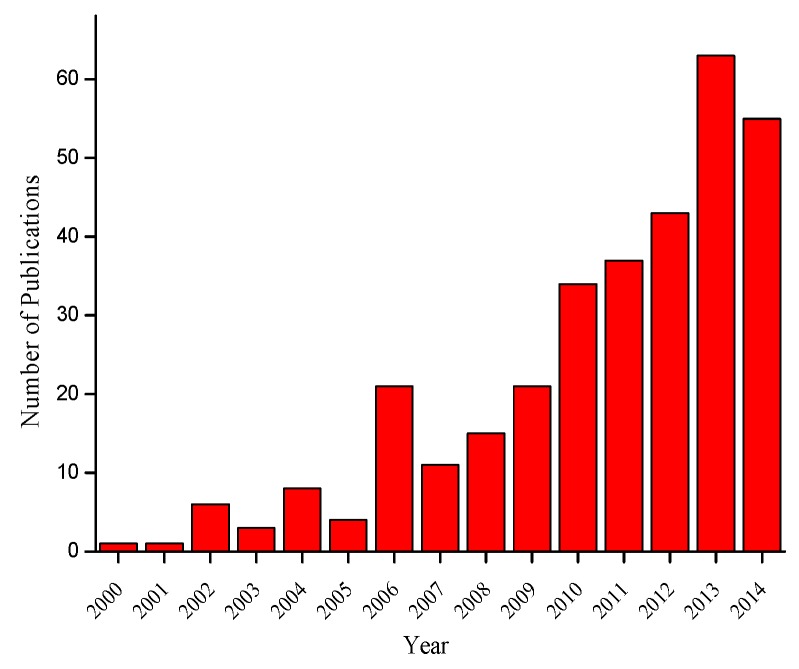
Number of publications which contains the terms “organic-inorganic” and “drug delivery” (ISI: Web of Science, accessed on 28 August 2015 [50]).

This review summarizes the use of silica based organic-inorganic hybrid materials for application in drug delivery systems. Figure 2 presents the number of publications related to the term “organic-inorganic” and “drug delivery” and shows the increase of these terms in recent years.

## 2. Applications of Organic-Inorganic Hybrids Materials in Drug Delivery

In this section, we mainly introduce silica based drug delivery systems, which showed effectivity when compared with traditional delivery systems. They present advantages such as: (a) ability to maintain the therapeutic range with minor variations; (b) prevention of local and systemic toxic effects; (c) allow to increase the plasma concentrations of substances with half-live short; and (d) allow the reduction of the number of daily doses, which may increase patient adherence to the treatment [5,6].

Due to the suitable mechanical, physical, chemical and optical properties of these materials, it is possible to develop multifunctional systems for drug delivery such as stimuli-responsive systems, biomaterials, film forming and others.

### 2.1. Stimuli-Responsive Systems

The polymers exhibit low critical solution temperature (LCST), so an increase of system temperature decreases its solubility in water due to changing the polarity and consequent predominance of hydrophobic interactions. Thus, these materials have attracted attention to the development of systems with the aim of releasing the drug after pathophysiological stimuli, providing optimal therapeutic levels and low toxicity [51,52].

Gao and co-workers [51] prepared a thermoresponsive hybrid polyvinyl alcohol (PVA)/Poly(*N*-isopropylacrylamide) (PNIPAAm) hydrogel using PVA and silica as matrix and porogenic agent, respectively. Rhodamine B (RB) was the drug model for the drug release study. The PVA pure and PVA/PNIPAAm were used to drug release study in predetermined temperatures (20 and 38 °C). It was concluded that PVA pure have not changed the release behavior in relation to different temperatures while the PVA/PNIPAAm influences significantly the release behavior, since RB is released slowly at 20 °C and RB release rate is increased at 38 °C.

Among stimuli-responsive systems, the pH responsive systems are more interesting since the human pH varies in several organs and target tissues. Therefore, in order to establish more effective treatments, Corma *et al.* [53] reported the use of organic-inorganic capped liposome with bioactive molecules (Doxorubicin) encapsulated into its aqueous cavity. This organic-inorganic shell could stabilize the internal liposomal phase and, consequently, isolate and protect the drug molecules. These liposomes system were developed with lecitine in chloroform/water. The formation of organic-inorganic phase around liposome is formed by reacting of pent-4-enoic acid and propen-2en-1-ol under reflux. The precursor pent-4-enoic acid allyl ester is bonded to silic units and results in ester-bridged silsesquioxane (BTEPAA). Other assay was performed to evaluate the amount of doxorubicin released into buffered aqueous solutions with different and controlled pH values (from pH 2.0 to 12.0, over 48 h). The authors observed that this system is stable at acid or neutral pH values and only at basic pH occurs the complete release of doxorubicin. They also analyzed, *in vitro*, the application of these systems into human glioma cells. The results suggest that the system achieved high values of cell mortality when compared with other drug delivery systems in the literature [53]. This system is interesting for the treatment of solid tumors due to the acidic extracellular pH environment.

Popat *et al.* [54] studied the pH responsive organic-inorganic system with the aim of improving therapeutic efficacy of ibuprofen. The system was prepared from covalent binding of positively charged polymer chitosan (Cs) onto phosphonate functionalized mesoporous silica nanoparticles (MSN) and MSN pure (without chitosan) was used as control group. In the drug release study, it was used two different pH media, pH 5 acetate buffer (simulating endosome pH) and pH 7.4 (simulating normal tissue pH). The authors observed that the MSN system, when pure, is able to reach the saturation of drug release in both pH after 4 h. The MSN coated by chitosan demonstrated different release rates. At pH 7.4, only 20% of ibuprofen was released and, at pH 5.0, it reached 90% after 8 h. The authors explain these behaviors by the fact that chitosan has low degradability and solubility at pH 7.4. These systems represent an important advance as pH-responsive nanocarrier and possess a drug modified release profile, increasing the effectiveness of treatment by reducing side effects [54].

Wan *et al.* [55] incorporated rhodamine B (RhB) into fluorescent pH sensing organic-inorganic system prepared from a mixture of random copolymers composed of *N*-(acryloxy)succinimide (NAS), oligo-(ethylene glycol) monomethyl ethermethacrylate (OEGMA), and 1,8-naphthalimide-based fluorescent pH-sensing monomer (NaphMA), which were anchored at the surface of mesoporous silica nanoparticles (MSN) via surface-initiated addition-fragmentation chain transfer (RAFT) polymerization. In this study, dithiothreitol (DTT) was used, which can cleave the disulfide linkage to open blocked nanopores, being the RhB release rate easily adjusted by adding different concentrations of DTT. It was verified that occurs an increase in the release of RhB with the increase of DTT concentration. The fluorescence properties were evaluated in various pH and showed that an increase intensity occurs in the range pH 5–7, which is suitable for monitoring intracellular pH changes and diseased tissues [55].

Organic-inorganic materials containing nanoparticles of magnetite (Fe_3_O_4_) were developed by Molina *et al.* [56] in order to achieve controlled drug delivery by magnetic field stimuli application. The system was composed by PEO (*O*,*O*′-bis(2-aminopropyl)-poly(propylene oxide) with 3-(isocyanatopropyl)-triethoxysilane, in a molar ratio of 1:2 and the drug model was sodium diclofenac. These authors analyzed the release of these systems without a magnetic field and with the application of an alternating magnetic field (0.25 T, 220 kHz). There was an increase in the amount of drug release from the system when the magnetic field was applied.

**Figure 3:**
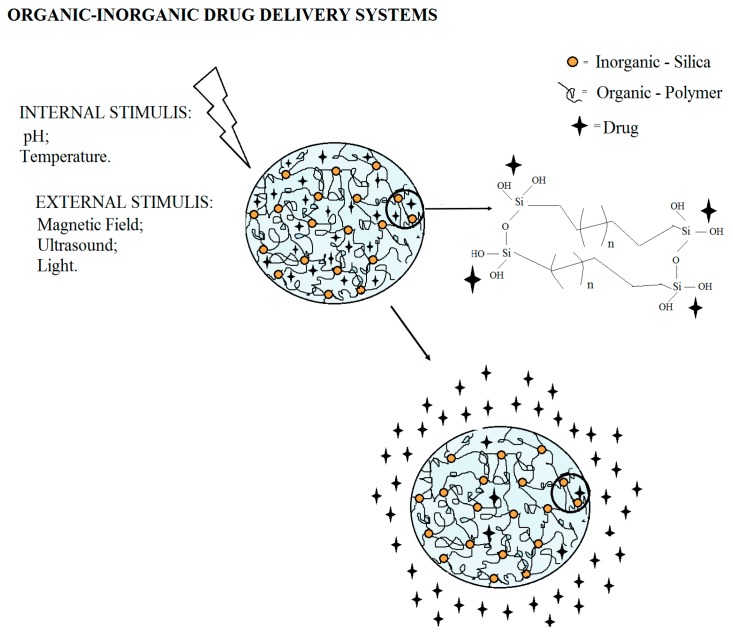
Schema of stimuli-responsive organic-inorganic materials for drug delivery systems (adapted from the study by Santilli *et al.*, 2009 [60]).

Figure 3 shows a scheme of the stimuli-responsive organic-inorganic material systems. The zoom shows a representative structural formula of the molecules of stimuli-responsive organic-inorganic materials and the possible sites of bonding with drugs. The release of the drug in this system can occur by external stimulus such as magnetic field, ultrasound, light, or internal stimulus as pH and temperature.

### 2.2. Implantable Biomaterials

**Figure 4:**
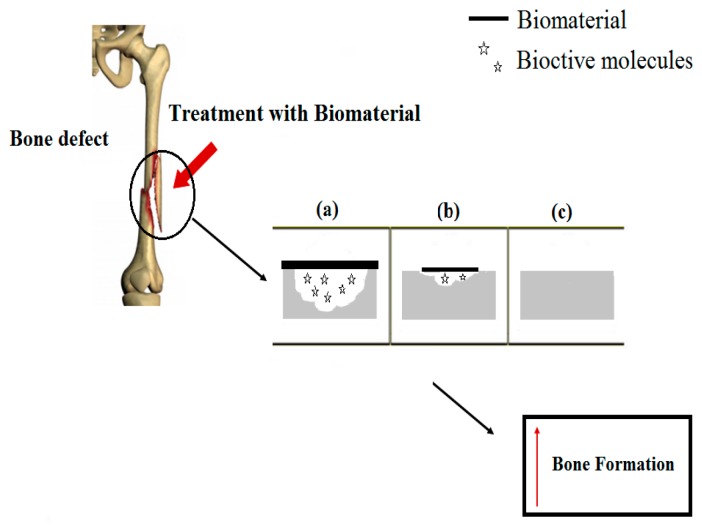
Bone defect repair using biomaterials: (**a**) the biomaterial releases bioactive molecules that interact with the bone cells; (**b**) it increases the quantity of bone tissue and occurs the degradation of the biomaterial, and finally; (**c**) occurs the regeneration of the tissue without presence of biomaterial.

An interesting example of DDS application is the use as biomaterials to repair the human body, being called third-generation biomaterials, since they are designed to interact with the biological environment. Their surface properties such as topography, surface charge, and all aspects related to their chemical composition surface are essential to obtain a positive response when these materials are in contact with biological tissue. This promotes cell adhesion, proliferation and differentiation [57]. Figure 4 shows the action of biomaterials in the bone defect repair.

Colilla *et al.* [58] studied the release of alendronate (biphosphonates widely used in the treatment of diseases with increased bone resorption) incorporated in the organic-inorganic systems, using Pluronic^®^ P123 as mesostructure directing agent. The template agent was dissolved in acid aqueous solution containing a certain amount of H_3_PO_4_ (85%, Aldrich) (P-SBA15 sample) or H_3_PO_4_ together with HCl (37%, Aldrich) (P-SBA15HCl sample). Tetraethyl orthosilicate was used as a silica source in the hybrid sample and also as pure silica mesoporous matrices to compare with hybrid matrices. They observed that the release rate of alendronate were higher in materials containing phosphorous (P-SBA15HCl and P-SBA15 sample) in relation to the control materials. The incorporation of phosphorous into the mesoporous silica provided the improvement of drug incorporation and sustained release compared to pure silica mesoporous matrices [58]. These systems can be an alternative to the treatment of diseases with increased bone resorption such as osteoporosis and osteolytic tumors. Moreover, they promote the decrease of bisphosphonates side effects incidence, since they exhibit low intestinal absorption and require a high dose to cause gastrointestinal disorders, chronic renal failure and others [59]. 

In the work conducted by Xue and Shi [61], gentamicin could be loaded in the organic-inorganic systems to obtain bone filling. The gentamicin is an antibiotic that can prevent a typical aggravation caused by biomaterials used in bone filling, called as osteomyelitis, which is a bone inflammation caused by pyogenic organisms. The gentamicin was incorporated into the system based on poly(d,l-lactide-*co*-glycolide) (PLGA)/mesoporous silica (PS) hybrid microspheres and mesoporous silica and drug loading efficiency were 45.6 and 22.4 wt %, respectively. The amount of drug released after 24 h reached ~77 wt % of mesoporous silica without PLGA, while PLGA/mesoporous silica reached ~41 wt %. It indicates that the polymeric phase acted as physical barrier and blocked the rapid release of drug during the first day. After 25 days, the release reached ~58 wt %, and was significantly slower than that exhibited by the mesoporous silica without PLGA, which reached ~90 wt % of release in 20 days. Xue and Shi suggested that in this stage occurs the control of the drug release from the mesoporous silica that was attached to the organic surface [61].

### 2.3. Film-Forming Materials

**Figure 5:**
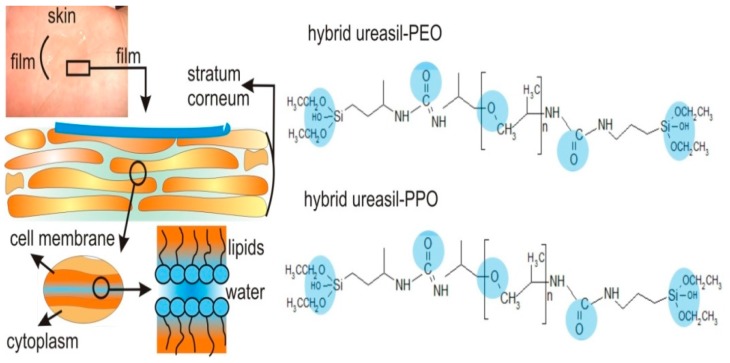
Molecules and biological interactions of ureasil-PEO and ureasil-PPO hybrid materials. Possible sites of bonding with the biological substrate and drugs are highlighted in the blue circles [64].

Film-forming materials represent an alternative to conventional Dermal Therapeutic Systems and Transdermal Therapeutic Systems because they offer the advantage of functional and comfortable treatment, since they are transparent, permitting the visualization of the wound and can be removed easier [62]. The use of organic-inorganic hybrid material named ureasil-polyether is an interesting example of film forming material described on the literature. Figure 5 shows the structural formulas of the molecules of ureasil-PEO and ureasil-PPO hybrid films and the possible sites of bonding with the biological substrate and drugs.

Souza *et al.* [63] evaluated the ureasil-polyether materials for this application. The adherence to the skin, the biocompatibility and the absence of occlusivity are required aspects for the application of a film-forming material in the protection of wounds from further injury or as transdermal drug delivery device. The system was prepared from the reaction of terminal amino-propyl groups of the functionalized poly(ethylene oxide) or poly(propylene oxide) with 3-(isocyanatopropyl)-triethoxysilane (Aldrich), using a molar ratio of 1:2. The results reveal that ureasil-polyether hybrids materials present semi-occlusive properties, enable the permeation of a portion of water vapor and retain the other part to hydrate the stratum corneum. It also exhibits biocompatibility and high adhesion force. Finally, this novel approach to drug-delivery systems presents suitable sensorial for dermal application.

Aiming to improve the characteristics of the ureasil-polyether films, Oshiro-Jr. *et al.* [64] conducted a study with the purpose to assess the influence of acid (hydrochloric acid) and basic (ammonium fluoride) catalyst in the hydrolysis and condensation reactions occurring on the formation and bioadhesion of ureasil-polyether films. The results reveal the nature of the catalyst influence on the speed and in the mechanism of film formation. Different mechanisms can change the speed of the drying process involved in the film formation, resulting in structural modifications that can be responsible to irregular film formations. The NH_4_F catalyst led to the formation of an irregular film, whereas HCl favored the formation of homogeneous and transparent films. This can be explained by the kinetics of the reactions promoted by the catalyst. The acid catalysis reaction is preceded by fast protonation of the substituents OR• or OH• attached to the Si, increasing the speed of reaction and resulting in a tendency to produce a more linear network, which results in more homogeneous films (15 mm wide and 0.5 mm thick) [64]. Thus, the catalyst NH_4_F did not form the film with the expected homogeneity within an acceptable time established by Schoeder *et al.* [62].

These hybrids are flexible, transparent and insoluble in water. Depending on the organic phase used, it can present hydrophilic or hydrophobic character. For this purpose, Oshiro-Jr. *et al.* [65] incorporated silver sulfadiazine into ureasil-polyether hybrids films to treat burn wounds using two polymers (1 hydrophilic (PEO) and 1 hydrophobic (PPO)) as the organic phase to assess the influence of hydrophilic/hydrophobic character in the rate of drug release. After five days of an *in vitro* experiment, it was observed that the amount of drug released from ureasil-PPO reached 10.6%, which was significantly lower when compared with ureasil-PEO that reached 24% of drug released. These results demonstrated that the drug release rate is dependent on the polarity characteristics of the matrices: more hydrophilic matrices present swelling capacity and favor the faster release of the drug.

Another example of these hybrid materials was the study conducted by Paredes *et al.* [66], which reveals the possibility of fine-tuning the release profile of drug (pramoxine 5% (*w*/*w*)) incorporated in ureasil-PEO containing chitosan polysaccharide. Chitosan is an abundant natural polysaccharide that presents biocompatibility and can be used in drug delivery systems to improve the rate of drug release. It was observed that ureasil-PEO reached 100% of pramoxine released after 180 min. On the other side, the presence of just 3% chitosan in the ureasil-PEO decreased the rate of release to 30% after 180 min. This could be because hydrophobic interactions occur between the chains of chitosan and the pramoxine molecules reducing the release rates.

### 2.4. New Approaches of DDS

Novel vehicles have been increasingly explored by researchers with the aim of improving transport of drugs through cell membranes. Several peptides have the ability to translocate through cell membranes and are called cell-penetrating peptides (CPPs). These CPP drugs attached to DDS is important in the protection of vehicle CPPs-drug from enzymatic action or chemical decomposition during storage or administration [67]. In this way, Gao *et al.* [68] developed organic-inorganic systems for the storage and CPP drug delivery using Pluronic^®^P127 and a Tetraethyl orthosilicateas silica source. The drug model used was fluorescein isothiocyanate (FITC) and octaarginine (R8) was used as a drug vector. In the first 36 hours of the drug release study, a very slow FITC-R8 release from mesostructured silica was observed. The amount of drug released after 36 h was accelerated and reached 85% in five days. The FITC-R8 pure and FITC-R8 with mesostructure were incubated with prostate cancer cells, demonstrating low toxicity to this cell line. In addition, this experiment proved the uptake of the FITC-R8 by the cells in the period of 96 h, whereas no signals were observed in the pure FITC-R8 [68].

Organic-inorganic materials containing ibuprofen were developed by Muñoz *et al.* [69], using hexadecyltrimethylammonium, dodecyltrimethylammonium surfactants with different chain sizes (16 and 12 carbon atoms, respectively). They were treated with 3-aminopropyl-triethoxysilane (AP-TES) to obtain both the aminopropyl-modified named MCM-4116a and MCM-4112a (method A). The MCM-4116b and MCM-4112b were synthesized by reaction of calcined MCM-41 and AP-TES (method B). These authors analyzed the formulations (MCM-4116a, MCM-4112a, MCM-4116b and MCM-4112b) by an *in vitro* release assay. The release rate was compared with original MCM-41 synthetized by Vallet-Regiet al. [70]. The ibuprofen release rate in the material MCM-4116a was identical to the original MCM-41 (50% released after 7 h of test). However, the release rate from the MCM-4116b was slower (50% released after 24 h of test). No differences were observed between materials with different chain sizes, and the material synthetized by the method B showed an improvement in the controlled-release properties due to the minor influence of the pore size [69].

Lin *et al.* [71] investigated the preparation and characterization of organic-inorganic materials based on polymethylmethacrylate (PMMA)/silica with various propyl methacrylate (MSMA) proportion, containing aspirin for biological applications. PMMA was already tested to repair bone-defects because it is biocompatible and bio-stable. The modification of PMMA with silica enables the bond to living tissues without fibrous capsule formation and the drug release control. The study of the release profile was performed with various concentrations of silica in the matrix of PMMA and various concentrations of MSMA with silica (10%) to allow the comprehension of the influence of organic-inorganic interface on drug release rate. The results showed that drug release rate increased with the increase of silica content, and the drug release rate decreased with the increasing of MSMA [71]. This fact is explained because the increased of silica in the sample created a major interfacial area (particle-polymer), which increased the water penetration and diffusion of drug.

Santilli *et al.* [60] suggested the potential application of ureasil cross-linked polyether materials for pharmaceutical application (contact lenses, patches and implantable drug delivery systems). In this study, four polymers were used, two of them being hydrophilic and the other two being hydrophobic and of different molecular weights, in order to verify the influence on the sodium diclofenac (DCFNa) released from the hybrid material. The precursors were prepared from a functionalized polyether, based on poly(ethylene oxide) (NH_2_-PEO-NH_2_) (*M*_w_ 500–1900 g·mol^−1^) (hydrophilic) (PEO), poly(propylene oxide) (NH_2_-PPO-NH_2_) (*M*_w_ 400–2000 g·mol^−1^)(hydrophobic) (PPO), and modified alkoxide, 3-(isocyanatopropyl)-triethoxysilane (IsoTrEOS) was added into the polymers(molar ratio of the polymer/alkoxide = 1:2). *In vitro* assays of drug release showed that DCFNa was released much faster from the hybrid matrices based on PEO. For the hydrophilic ureasil-PEO matrices, the drug molecules can easily diffuse into the release medium through the free volume of the swollen network. Under these conditions, the diffusion of dissolved drug molecules to the liquid environment is a time–dependent process that can be predicted by assuming a pseudo first–order kinetic. On the other hand, the lower hydrophilicity of the hybrid matrices PPO hinders the hydration and dissolution of the drug and its diffusion into the medium [60].

**Figure 6:**
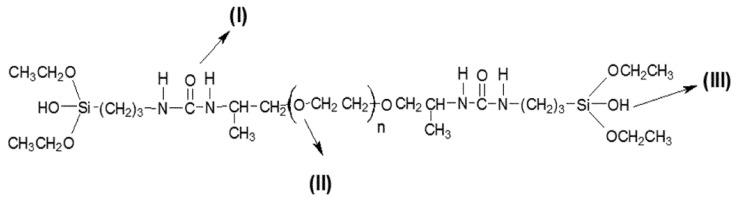
Possible links between sites of hybrid material and molecules (I, II and III) [60].

This same research group demonstrated that these materials have the ability to incorporate other compounds such as the antitumoral cisplatin. The release profile of these compounds follows the same mechanism described for DCFNa [72]. This versatility is due to the important existing groups in the molecule. Figure 6 presents the links between sites of hybrid material and molecules (I, II and III). These ureasil-polyether materials have two types of electronegative bonding sites, the oxygens of ether type (II) in the polymer chain, silanol group (III) and also an electropositive binding site (NH group (I)) of the urea grouping, which can bind electronegative molecules. Lopes *et al.* [73] revealed through Raman data that these drug–matrix interactions involve mainly the amine and carbonyl groups of the DCFN, a molecule through the formation of a new hydrogen bonding structure with urea groups present at the end of PEO chains. Because of this drug–matrix hydrogen bonding, the total amount of DCFN delivered decreases as the proportion of ureasil linkages increases, *i.e.*, by lowering the PEO chain length from PEO1900 to PEO500.

Herculano *et al.* [74] showed that the release of nitric oxide (NO) could be improved when incorporated in ureasil-PPO (*M*_w_ 400 g·mol^−1^). NO is a free radical indicated for the treatment of blood vessel protection. This action is associated with (i) maintenance of vascular tone; (ii) blood pressure regulation; (iii) prevention of platelet aggregation; and (iv) antioxidative effect [75,76]. However, the detection of NO is a challenge because it is highly reactive, which means it has short lifetimes in living organisms, depending on the environment, which could vary from milliseconds to minutes. Thus, the authors use encapsulation of iron(II)-diethyldithiocarbamate (FeDETC) in various matrices for either the measurement of NO or its release due to the affinity between NO and the iron complexes, allowing the use of electron paramagnetic resonance (EPR) to radical detection [77]. The studies showed that it was possible to detect the release of NO after 40 days [74]. This system is an important advance in the development of DDS containing NO for the treatment of heart diseases.

The discussion made in the research papers presented the above account for the innovation that the organic-inorganic hybrid materials represent. In brief, these materials are able to incorporate hydrophilic and lipophilic drugs, which could be used by consumer/patients by various pathways.

The rate of release can also be controlled, depending on the composition of the organic-inorganic hybrid system. Table 1 helps in the comprehension about the influence of the composition in the time of drug release.

## 3. Conclusions

Hybrid materials of the organic-inorganic type represent a new alternative to conventional drug delivery systems. Controlled release offers many advantages over conventional release forms such as high release efficiency, precise control of the dosage form for prolonged periods and decreased toxicity. Among the organic-inorganic hybrid materials, silica bases stand out due to the high stability of the Si–C bond. Moreover, the Si–O–Si bond confers transparency, thermal stability and mechanical resistance. The versatility of these materials can be explored for the development of several kinds of controlled drug delivery devices like implants, adhesives, masks, microspheres and others. These devices are a new approach for treatment of several diseases; however, more studies are necessary to evaluate their biocompatibility.

## Figures and Tables

**Table 1:** Composition and time of release of drug to selected organic-inorganic systems.

Organic-Inorganic System Composition	Drug	Time of Released of Drug	Comments	Reference
PVA/PNIPAAm	Rhodamine B	16 h = ± 95% at 38 °C 16 h = ± 70% at 20 °C	Temperature influences in the release behavior	[51]
BTEPAA	Doxorubicin	48 h = ± 10% in pH ≤ 9 and ± 95% in pH ≥ 9.	pH responsive systems	[53]
Cs/MSM	Ibuprofen	4 h = ± 20% in pH 7.4 and ± 90% in pH 5.	pH responsive systems	[54]
NAS/NAphMA/OEGMA/MSM	Rhodamine B	4 h = ± 95% with 20 mM DDT 4 h = ± 5% without DDT	Fluorescence properties	[55]
PEO/3-(isocyanatopropyl)-triethoxysilane/Magnetite	Sodium diclofenac	4 h = ± 95% with the application magnetic and 80% without magnetic field (0.250 T, 220 kHz)	Stimulis with Magnetic field	[56]
Pluronic/Tetraethyl orthosilicate/P_2_O_5_/H_3_PO_4_	Alendronate	4 h = 25 µg·m^−2^ to material without P_2_O_5_ and 87 µg·m^−2^ to material with P_2_O_5_	Implantable systems	[58]
PLGA/PS	Gentamicin	24 h = 77% PLGA pure and 41% PLGA/PS. 25 days = 90% PLGA and 58% PLGA/PS	Implantable Systems	[61]
PEO/3-(isocyanatopropyl)-triethoxysilane/Chitosan	Pramoxine	3 h = ± 100% without chitosan and 30% with 3% of chitosan	Film-forming systems	[66]
Pluronic(127)/CPPs/Tetraethyl orthosilicateas	FITC/octaarginine	5 days = 85%	New devices of DDS	[68]
